# Severe bradycardia at the termination of seizure during electroconvulsive therapy

**DOI:** 10.1186/s40981-020-00389-6

**Published:** 2020-10-16

**Authors:** Yuji Kadoi, Minoru Michizaki, Takanari Saito, Jo Ota, Shigeru Saito, Tatsuo Sameshima

**Affiliations:** 1 Gunma University Hospital, 3-39-22 Showa-Machi, Maebashi, Gunma 371-8511 Japan; 2 Department of Anesthesiology, Gunma University School of Medicine, 3-39-15, Showa-machi, Maebashi, Japan; 3 Department of Psychiatry, Minkodo Aburayama Hospital, 5-6-37, Noke, Sawaraku, Fukuoka, Japan

**Keywords:** Severe bradycardia, Electroconvulsive therapy, Autonomic nerve system

## Abstract

**Background:**

Few cases of asystole or severe bradycardia occurring after the termination of seizure in the third phase with the dominance of parasympathetic nervous system activity during electroconvulsive therapy (ECT) have been reported. We describe a case of severe bradycardia occurring at the termination of seizure.

**Case presentation:**

The patient had been diagnosed with bipolar disorder more than 9 years earlier. No adverse hemodynamic events had been observed in over 100 sessions of ECT performed during a 9-year period. ECT was usually induced by propofol and suxamethonium. On this ECT, the heart rate gradually decreased before seizure termination, and severe bradycardia (5–6 beats/min) was identified lasting 15–20 s. Atropine administration immediately before electrical stimulus prevented any further bradycardia during the next session of ECT.

**Conclusions:**

This case report indicates that attention should be paid to adverse cardiac events related to autonomic nerve activity even before such events occur during ECT.

## Background

Electroconvulsive therapy (ECT) is performed worldwide as an effective therapeutic option to relieve symptoms of psychiatric depression [1]. During ECT, parasympathetic discharges immediately after electrical stimulation suppress the heart rate (HR) [1, 2]. In some patients, bradycardia is severe, and temporary asystole can be observed [2, 3]. However, this first phase is completed within 1 min, and the ensuing second phase of sympathetic activation induces tachycardia and elevated blood pressure, with drastic hemodynamic changes lasting > 10 min after electrical stimulation [1, 4].

Suzuki et al. [5] showed a triphasic change in cardiac autonomic activity from parasympathetic to sympathetic and back to parasympathetic dominance after ECT stimulus onset. That report suggested that asystole or severe bradycardia could occur after seizure termination in the third phase, with a dominance of parasympathetic activity [5]. However, most reports have only described asystole or severe bradycardia immediately after electrical stimulation in the first phase of parasympathetic dominance [6–10]. To date, there have been few reports of asystole after seizure termination in the third phase of parasympathetic dominance during ECT [11–13].

We describe a case in which severe bradycardia occurred before seizure termination in the third phase of parasympathetic dominance.

## Case presentation

A 66-year-old, 57 kg, woman with bipolar disorder was scheduled for maintenance ECT. She had been diagnosed with bipolar disorder over 9 years earlier. She had undergone 12 sessions of ECT under general anesthesia in our hospital. Propofol and suxamethonium were used for general anesthesia in every ECT procedure. ECT was effective against the bipolar disorder, and she had achieved excellent clinical response with maintenance ECT every 2–3 months over 9 years in our hospital. With over 100 sessions of ECT during the 9 years, no adverse hemodynamic events of note (including bradycardia) had been observed, and no atropine had been administered at any session of ECT in our hospital. In addition, ECT procedures over the previous 2 months showed no noteworthy events, including asystole or bradycardia. Five years earlier, transthoracic echocardiography had been performed because of abnormalities on electrocardiography (ECG) in the form of incomplete right bundle branch block (IRBBB). No abnormalities were identified on echocardiography, with no asynergy of the left ventricle (LV) and an LV ejection fraction of 60–65%.

At this time, treatment with maintenance ECT was planned to be continued as usual, along with pharmacotherapy comprising oral olanzapine at 5 mg/day.

For the ECT session in which the event occurred, no premedication was administered. Pre-intervention ECG indicated normal sinus rhythm with IRBBB. No abnormality of ST-T change was observed. A cardiologist performed cardiac examinations and found no issues requiring consideration. No abnormalities were evident from pre-intervention laboratory analyses, including serum potassium concentration (3.6 mEq/L). Oral olanzapine at 5 mg was continued on the day of ECT.

In the operating room, baseline blood pressure (BP) was 161/94 mmHg, HR was 77 beats/min, and peripheral oxygen saturation was 96% under room air.

Anesthesia was induced using propofol at 1.0 mg/kg, and then 0.6 mg of suxamethonium was administered. After the induction of anesthesia, hyperventilation (end-tidal carbon dioxide, approximately 30 mmHg) was achieved by manual mask ventilation with 100% oxygen. When muscular relaxation was judged as adequate, bilateral electrodes were placed, and the electrical stimulus for ECT was delivered using a Thymatoron system IV® (Somatics; LLC, Lake Buff, IL) via bifrontal-temporal electrode placement (electric current, 0.92 A; frequency, 40 Hz; stimulation duration, 6.3 s).

**Fig. 1:**
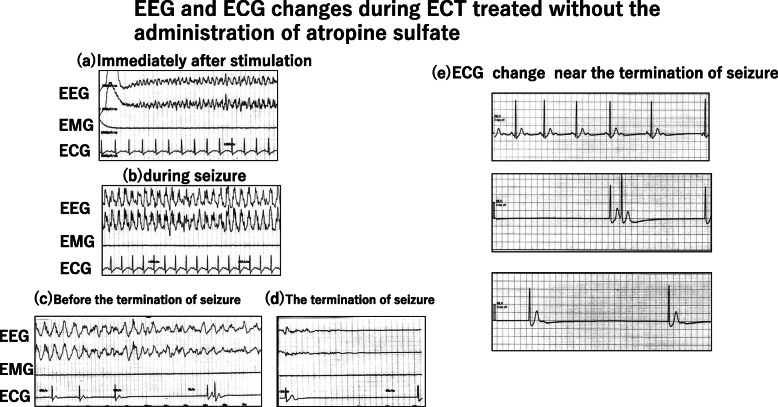
EEG and ECG changes during ECT without administration of atropine sulfate. **a** Immediately after stimulation. **b** During seizure. **c** Before seizure termination. **d** Seizure termination. Severe bradycardia is observed from before seizure termination until seizure termination (**c**, **d**)

Immediately after electrical stimulation, systolic BP increased from 144 to 188 mmHg, and HR increased from 81 to 151 beats/min (Fig. 1a, b).

Tonic-chronic seizures were induced (motor seizures, 55 s; electroencephalographic (EEG) seizures, 73 s) (Fig. 1b, c).

Shortly before seizure termination, HR gradually decreased, and severe bradycardia (5–6 beats/min) was identified for 15–20 s (Fig. 1c, d). The patient was administered 0.5 mg of atropine sulfate, and HR quickly normalized to 78 beats/min. Recovery from ECT was uneventful, with no further episodes of asystole or other arrhythmias. A consulting cardiologist indicated that no further evaluation was required and recommended conservative management.

**Fig. 2:**
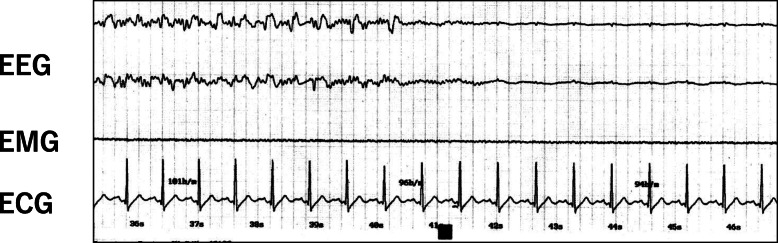
EEG and ECG changes near seizure termination with administration of atropine sulfate before electrical stimulation. Administration of atropine sulfate prevented severe bradycardia during seizure termination

For the next ECT procedure, 0.5 mg of atropine sulfate was infused immediately before the electrical stimulus, and no adverse hemodynamic changes, asystole, or severe bradycardia were found during ECT (Fig. 2).

## Discussion

The current case showed severe bradycardia, not immediately after the onset of electrical stimulation, but near seizure termination, which was considered to be in the third phase of parasympathetic dominance.

With regard to changes in the autonomic nervous system, three phases of activity occur during ECT [1, 5]. ECT initially produces parasympathetic-induced bradycardia lasting 10–15 s through electrical stimulation of the nucleus of the vagal nerve, followed immediately by a more prominent sympathetic response that results in transient tachycardia and hypertension. This sympathetic response continues until the seizure stops, when the parasympathetic nerve system is predominant.

In clinical situations of the sympathetic dominance in the second phase, serum levels of norepinephrine and epinephrine increase 3- to 15-fold, and neuroendocrine response includes prolactin, beta-endorphin, adrenocorticotropic hormone (ACTH), cortisol, and growth hormone [4]. Exaggerated increases in norepinephrine and epinephrine or other hormones induced by electrical stimulation may easily mask the third phase of parasympathetic response in clinical situations and could provoke arrhythmias such as bradycardia or asystole in the third phase of parasympathetic response.

Some cases of asystole or severe bradycardia during ECT have been reported [6–10]. In most of these case reports, asystole or severe bradycardia was observed immediately after electrical stimulation in the first phase of parasympathetic nerve system activity. Few reports have shown asystole or severe bradycardia in the third phase of parasympathetic nerve system activity [11–13].

Bryson et al. [11, 12] reported two cases of vagally mediated postictal systole during ECT. Takagi et al. [13] showed a case of asystole occurring a few seconds after electrical stimulus without antihypertensive medication. They speculated that this asystole was due to the pathological reflex in carotid sinus syndrome. Suzuki et al. [5] examined the time course of autonomic changes during ECT using HR variability. They showed that significant reductions in averaged HR over 3 beats just after ECT were attributable to parasympathetic dominance in the first phase. The ratio of low-frequency (LF) power to high-frequency (HF) power offers an index of sympathetic activity and is significantly increased at 30 to 80 s after stimulus onset, whereas HF power (as an index of parasympathetic activity) increased significantly at 80 to 130 s range after stimulus onset, reflecting sympathetic activation in the second phase and parasympathetic activation in the third phase, respectively. These results confirmed a triphasic change from parasympathetic to sympathetic to parasympathetic cardiac autonomic activity after ECT. In our case, atropine sulfate was administered before the next ECT procedure, and no bradycardia occurred. This provides some degree of evidence that severe bradycardia after seizure termination was attributable to the exaggerated parasympathetic nerve system activity in the third phase of parasympathetic dominance during ECT.

It remains controversial whether 1 anti-cholinergic agent such as atropine or glycopyrrolate should be used to prevent bradycardia immediately before electrical stimulation in all cases [1]. Atropine sulfate, injected to prevent the adverse effects of parasympathetic-induced bradycardia, can conversely induce myocardial ischemia. However, Mokriski et al. [14] reported that intravenous administration of 0.6 mg of atropine before anesthesia induction could decrease the frequency of premature atrial contractions and bradycardia and increase the frequency of tachycardia after ECT.

Why severe bradycardia was observed on this occasion remains uncertain, as this patient had previously been treated with ECT over 100 times without any adverse events. One possible mechanism is that the balance between sympathetic and parasympathetic activity might alter with age. Yamaguchi et al. [15] showed more profound ECG changes after electrical stimulus in elderly patients compared with those in young patients. Vagally mediated parasympathetic activity may increase with age. Another possibility is that parasympathetic modulation may be restored with the improvement of depressive symptoms [5]. Because her parasympathetic modulation was restored by previous medical therapies such as ECT and oral medication, parasympathetic overactivity after ECT may have been present for this event.

This case report indicates that attention should be paid to adverse cardiac events related to autonomic nerve activity even before such events occur during ECT.

## Conclusions

We describe a case in which severe bradycardia occurred around seizure termination in the third phase of parasympathetic dominance during ECT. This case report indicates that attention should be paid to adverse cardiac events related to autonomic nerve activity even before such events occur during ECT.

## Data Availability

Not applicable

## Abbreviations

ECT: Electroconvulsive therapy
ECG: Electrocardiography
IRBBB: Incomplete right bundle branch block
EEG: Electroencephalography
HR: Heart rate
BP: Blood pressure
ACTH: Adrenocorticotropic hormone
LF: Low frequency
HF: High frequency
